# Supplementary material for: Socioeconomic status and risk of gestational diabetes mellitus among pregnant women in Zunyi, China
Source: PeerJ. 2025 Aug 28;13:e19782. doi: 10.7717/peerj.19782 (PMC12399077; doi:10.7717/peerj.19782)
Supplement: Supplemental Information 2 [file peerj-13-19782-s002.docx]

**Highlights**

1. This study aimed to explore the relationship between socio-economic status (SES) and gestational diabetes mellitus (GDM) to address gaps in existing research.
2. We aimed to investigate how education level, occupation, and household income impact the prevalence of GDM.
3. We found no significant association between education level and GDM, but occupation and household income significantly influenced GDM risk.
4. Our findings suggest that occupation and household income are stronger predictors of GDM than education level, providing new insights for public health interventions.
